# Measures of Neural Similarity

**DOI:** 10.1007/s42113-019-00068-5

**Published:** 2019-12-02

**Authors:** S. Bobadilla-Suarez, C. Ahlheim, A. Mehrotra, A. Panos, B. C. Love

**Affiliations:** 1 Department of Experimental Psychology, University College London, 26 Bedford Way, London, WC1H 0AP UK; 2 Department of Geography, University College London, Gower Street, London, WC1E 6BT UK; 3 Department of Statistical Science, University College London, Gower Street, London, WC1E 6BT UK; 4 The Alan Turing Institute, 96 Euston Road, London, NW1 2DB UK

**Keywords:** Neural similarity, Neural coding, Machine learning, fMRI

## Abstract

One fundamental question is what makes two brain states similar. For example, what makes the activity in visual cortex elicited from viewing a robin similar to a sparrow? One common assumption in fMRI analysis is that neural similarity is described by Pearson correlation. However, there are a host of other possibilities, including Minkowski and Mahalanobis measures, with each differing in its mathematical, theoretical, and neural computational assumptions. Moreover, the operable measures may vary across brain regions and tasks. Here, we evaluated which of several competing similarity measures best captured neural similarity. Our technique uses a decoding approach to assess the information present in a brain region, and the similarity measures that best correspond to the classifier’s confusion matrix are preferred. Across two published fMRI datasets, we found the preferred neural similarity measures were common across brain regions but differed across tasks. Moreover, Pearson correlation was consistently surpassed by alternatives.

**Electronic supplementary material:**

The online version of this article (10.1007/s42113-019-00068-5) contains supplementary material, which is available to authorized users.

## Introduction

Detecting similarities is critical to a range of cognitive processes and tasks, such as memory retrieval, analogy, decision-making, categorization, object recognition, and reasoning (Aly et al. 2013; Bracci and de Beeck 2016; Coutanche and Thompson-Schill 2014; Goldstone 1994; Markman et al. 2006; Medin et al. 1993; Palmeri and Gauthier 2004; Tyler et al. 2000). Key questions for neuroscience include which measures of similarity does the brain use and do similarity computations differ across brain regions and tasks. Whereas psychology has considered a dizzying array of competing accounts of similarity (Ennis et al. 1988; Gentner and Markman 1997; Hahn et al. 2003; Krumhansl 1978; Pothos et al. 2013; Tenenbaum and Griffiths 2001; Tversky 1977), research in neuroscience usually assumes that Pearson correlation captures the similarity between different brain states (Davis and Poldrack 2013; Davis et al. 2014; Kriegeskorte et al. 2008a; Kriegeskorte et al. 2008b; LaRocque et al. 2013; Nili et al. 2014; Weber et al. 2009; Xue et al. 2010); albeit, not all (Gardella et al. 2018; Nili et al. 2014; Ramirez et al. 2014; Soucy et al. 2009; van Rossum 2001).

Of course, when evaluating whether the brain favors certain measures of similarity, any conclusions are with respect to the chosen data sets and dependent measures. This caveat is shared with other endeavors, such as determining which algorithm the brain uses for category learning. Although category learning models are typically selected based on a set of behavioral studies, model comparison can also be done on the basis of brain imaging data (Mack et al. 2013). Here, we select an abstract measure of similarity based solely on brain data, in particular fMRI data. Although our methods could equally apply to other measures of neural activity, such as single-unit recording or EEG data, we focus on fMRI because of its ability to localize activity from a number of brain regions simultaneously and demonstrations that it can recover similarity spaces despite the method’s limitations, which itself can be illuminating of the underlying neural computations (Guest and Love 2017). We alert the reader that, like any investigation that aims to bridge levels (e.g., from brain measure to abstract similarity computation), the chosen data sets (e.g., the tasks, the stimuli, the dependent measures) play a role in shaping the results. With this caveat, we proceed and evaluate similarity measures that operate over fMRI voxels.

On the face of it, it seems unlikely that the brain would use a single measure of similarity across regions and tasks. First, across regions, the signal and type of information represented can differ (Ahlheim and Love 2018; Bracci and de Beeck 2016; Diedrichsen et al. 2011), which might lead the accompanying similarity operations to also differ. Second, task differences, such as those that shift attention (Braunlich and Love 2018; Mack et al. 2013; Mack et al. 2016), lead to changes in the brain’s similarity space which may reflect basic changes in the underlying similarity computation. Our interest is in describing similarity computations that could, in principle, be used for behavioral output, focusing on a necessary but not sufficient condition for producing behavior from neural representations. Admittedly, similarity operations can be defined not only over voxel vectors but as attentional weights on stimulus dimensions (Mack et al. 2013; Mack et al. 2016), but we will not address this phenomena here. Outside neuroscience, it is common to use different similarity measures on different representations. For example, in machine learning, Euclidean measures are often used to determine neighbors in image embeddings whereas cosine similarity is more commonly used in natural language processing (Mihalcea et al. 2006).

In this contribution, we developed a technique to address two theoretical goals. The first goal was to ascertain whether the similarity measures used by the brain, as measured by fMRI, differ across regions. The second goal was to investigate whether the preferred measures differ across tasks and stimulus conditions. Our broader aim was to elucidate the nature of neural similarity. To do this, we propose using the confusion matrix of a best-performing classifier to evaluate similarity measures, with the classifier being chosen by a best decoding accuracy criteria.

Previous studies have adopted different similarity measures to relate pairs of brain states such as Pearson correlation or the Mahalanobis measure, measures commonly chosen for representational similarity analysis (RSA) (Allefeld and Haynes 2014; Haxby et al. 2011; Kiani et al. 2007; Kriegeskorte et al. 2008a). However, the basis for choosing one measure over another is not always clear. The choice of measure induces a host of assumptions, including assumptions about how the brain codes and processes information. While all the measures considered operate on two vectors associated with two brain states (e.g., the BOLD response elicited across voxels when a subject views a truck vs. a moped), the operations performed when comparing these two vectors differ for each similarity measure.

### Families of Similarity Measures

**Fig. 1:**
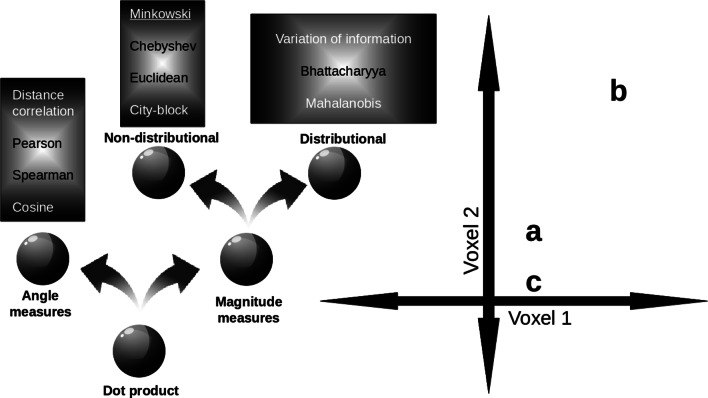
Families of similarity measures. (left panel) Similarity measures divide into those concerned with angle vs. magnitude differences between vectors. Pearson correlation and Euclidean distance are common angle and magnitude measures, respectively. The magnitude family further subdivides according to distributional assumptions. Measures like Mahalanobis are distributional in that they are sensitive to co-variance such that similarity falls more rapidly along low variance directions. (right panel) The choice of similarity measure can strongly affect inferences about neural representational spaces. In this example, stimuli **a**, **b**, and **c** elicit different patterns of activity across two voxels. When Pearson correlation is used, stimulus **a** is more similar to **b** than to **c**. However, when the Euclidean measure is used, the pattern reverses such that stimulus **a** is more similar to **c** than **b**

To better understand these assumptions and their importance, we organize common measures of similarity, many of which are used in the neuroscience literature, into three families (see Fig. 1, left side). The most basic split is between similarity measures that focus on the angle between vectors (e.g., Pearson correlation or cosine distance) and measures that focus on differences in vector magnitudes. The latter branch subdivides between distributional measures that are sensitive to covariance across vector dimensions (e.g., Mahalanobis) and those that are not (e.g., Euclidean). Of course, there are uncountably infinite similarity measures one could choose to assess; the goal here is to compare common measures that can discriminate between different computations of interest as organized by these families of measures with focus on angle, magnitude, and distributional properties.

The choice of similarity measure can shape how neural data are interpreted, leading to inferences on the underlying computation. Consider the right panel in Fig. 1. In this example, the neural representation of object **a** is more similar to that of **b** than **c** when an angle measure is used, but this pattern reverses when a magnitude measure is used.

Unlike the other measures, distributional measures are anisotropic, meaning the direction of measurement is consequential.^1^ Examples of such measures are variation of information, Mahalanobis, and Bhattacharyya measures. These measures consider the covariance between dimensions in voxel space, which implies that the direction along which the measurement is made will impact the measurement itself.

The choice of similarity measure reflects basic assumptions about the nature of the underlying neural computation. For example, Pearson correlation (a common measure for neural similarity in fMRI, e.g., Davis and Poldrack (2013); Davis et al. (2014); Kriegeskorte et al. (2008a, 2008b); LaRoque et al. (2013); Nili et al. (2014); Weber et al. (2009); Xue et al. 2010) assumes that overall levels of voxel activity are normalized and that each voxel independently contributes to similarity, whereas Minkowski measures assume similarity involves distances in a metrical space instead of vector directions. Furthermore, the Mahalanobis measure expands on both Minkowski and Pearson by assuming that the distributional pattern of voxel activity is consequential. Non-distributional measures, like Pearson correlation, require less data than distributional measures since they are not concerned with estimating a covariance matrix. Contrariwise, distributional measures will be biased to operate over vectors with lower dimensionality; this is covered in the “Materials and Methods” section below where our feature selection procedure levels the playing field for all measures. This also hints as to whether neural computations are more or less spatially localized or if they are integrating information over longer time periods (i.e., a covariance matrix represents this longer time period since it requires more information from more stimulus observations). As alluded to here, finding a good description of the brain’s similarity measure is as important as finding an appropriate coordinate system for neural stimulus representation; these goals are in fact equivalent — similarity measures can be seen as doing implicit coordinate transforms.

Knowing which similarity measure best describes the brain’s operation could illuminate the nature of neural computation at multiple levels of analysis. For example, if a brain region normalized input patterns for key computations, then Pearson correlation might have superior descriptive power than the dot product. At a lower level, such a result would be consistent with mutually inhibiting single cells (Heeger 1992). On the other hand, if the brain matches to a rigid template or filter (e.g., Brunelli and Poggio (1993)), then the Euclidean measure should provide a better explanation for neural data.

To identify which similarity measures are used by the brain requires addressing a number of challenges. One challenge is to specify a standard by which to evaluate competing similarity measures. Related work in psychology and neuroscience has relied on evaluating against verbal report. However, such an approach is not suited to our aims because we are interested in neural computations that may differ across brain regions and which may not be accessible by verbal report or introspection.

Instead, we rely on a decoding approach to assess the information latent in a brain region. The intuition is that brain states that are similar should be confusable in decoding. For example, a machine classifier may be more likely to confuse the brain activity elicited by a bicycle with that by a motorcycle than a car. In this fashion, we can evaluate competing similarity measures on a per region basis in a manner that is not constrained by verbal report. The insight that similarity is intimately related to confusability has a long and rich intellectual history (Shepard 1964; Spence 1952; Pavlov and Anrep 2003) though has not yet been considered to evaluate what makes two brain states similar.

### Discrimination of Similarity Measures

Our method for distinguishing the similarity measure used by the brain involves two basic steps:

1. For each ROI, compute a pairwise confusion matrix^2^ using a classifier. For each ROI, also compute a similarity matrix for each candidate similarity measure.
2. For each similarity measure, correlate its similarity matrix with the confusion matrix using Spearman correlation to avoid scaling issues.

The better a similarity measure characterizes what makes two brain states similar, the higher its Spearman correlation with the confusion matrix should be. This analysis uses the confusion matrix as an approximation of what information is present in a brain region (more on this below).

**Fig. 2:**
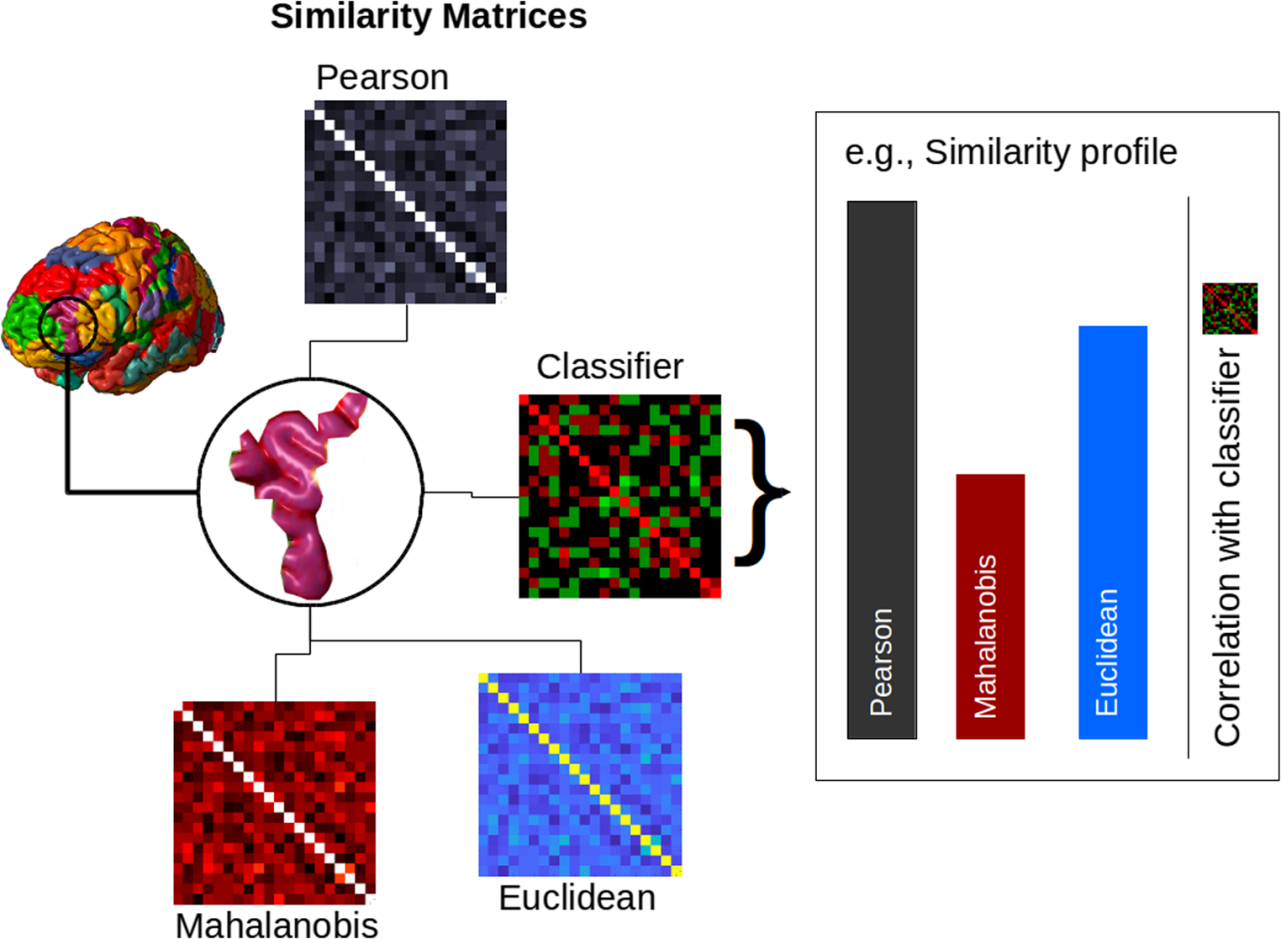
Evaluating the similarity profile for a ROI. The confusion matrix from a classifier is used to approximate the information present in the ROI. The similarity matrix from each similarity measure is correlated with this confusion matrix (i.e., the classifier matrix in the figure). The pattern of these correlations (i.e., the performance of the various similarity measures) is the similarity profile for that ROI. Similarity profiles can be compared between ROIs, both within and between datasets (see “Materials and Methods” section for more details)

The matrices for each similarity measure were optimized to maximize the Spearman correlation with the confusion matrix by performing feature selection on voxels (see Fig. 2). See the SI (Supplemental Information) for details on the similarity measures. Importantly, to understand the results, some similarity measures (i.e., Mahalanobis and Bhattacharya) that estimate covariance matrices are tagged according to the type of regularization used, with (d) for keeping only the diagonal entries and (r) for Ledoit-Wolf shrinkage.

We considered all 110 regions of interest (see SI for a list of the 110 regions) from the Oxford-Harvard Brain Atlas (provided with FSL, Jenkinson et al. (2012)) for two previously published datasets. One dataset was from a study in which participants viewed geometric shapes (GS) (Mack et al. 2013), and the other dataset was from a study in which participants viewed natural images (NI) (Bracci and de Beeck 2016). For each dataset, we determined the top 10 ROIs for decoding accuracy (cf. Bhandari et al. (2018)). The union of these top ROIs provided 12 ROIs that were considered in subsequent analyses (see SI).

### Lower Confusability as Information Gain

As mentioned above, our proposed method involves approximating brain state information with a classifier. Subsequently, we use this approximation to assess an array of similarity measures. The motivation for using a classifier to approximate information in a brain state arises from an information theoretic perspective. For example, suppose one’s prior assumption is that two stimuli are equally likely, which corresponds to random guessing or maximal entropy (1 bit). If a probabilistic classifier with the same prior is applied to the stimulus and approaches 100% accuracy, then the information gain approaches 1 bit. Formally, one can measure the Kullback-Leibler (KL) divergence (a continuous, non-saturating measure) between a prior distribution *p* (centered at 0.5) and an updated distribution *q* defined by the classifier’s output. To be more specific, we could model the prior as a binomial distribution with parameter *p* and the updated distribution as another binomial with parameter *q*. With a suitable prior distribution for the classifier, the KL divergence is always defined and enables a computable measure of brain state information. Thus, KL divergence, or information gain, will be inversely proportional to confusability as measured by the classifier. Of course, in practice, machine classifiers do not reach close to 100% accuracy with fMRI data for the types of discriminations that we consider. The point is that decoding and measuring available information in a brain state are intimately linked. This further justifies the black box approach to choosing a classifier with the highest decoding accuracy to approximate ground truth confusability in the brain. Our hope is that treating the classifier selection process as a black box will reduce the bias for choosing the best-performing similarity measure.

### Classification is not Similarity

Although it should be clear to cognitive scientists of all varieties that similarity and classification are conceptually distinct (see Goldstone 1994), it may not be as apparent to some neuroscientists whose focus is elsewhere. To view similarity and classification as one in the same would be akin to viewing any operation in which similarity could be relevant, such as memory retrieval, as synonymous with similarity (Medin et al. 1993).

Mathematically, the domain and range of similarity and classification functions are distinct. Similarity takes as its domain (i.e., input) two states and its range (i.e., output) is a scalar value (i.e., the similarity). Notice that similarity can apply to any two states, irrespective of class membership. A similarity function does not need to be “trained” and “tested” on a particular discrimination, but instead can apply broadly. Thus, new classes can be evaluated without training. In contrast, a classification function takes as its domain (i.e., input) items drawn from a predetermined set of classes and its range (i.e., output) is a nominal value indicating the class membership of the item. A classifier is trained on items from the contrasting classes and tested only on items drawn from these same distributions. The existence of special cases in which there is a close relationship between a particular similarity measure and classifier is not a valid argument that similarity and classification are one in the same in any general sense. It is true that some classifiers may rely on similarity as an internal operation, but such examples do not equate the categories. Likewise, some similarity functions may require more information than others (e.g., estimating a covariance matrix), but this does not limit the applicability of that similarity function to new classes.

**Fig. 3:**
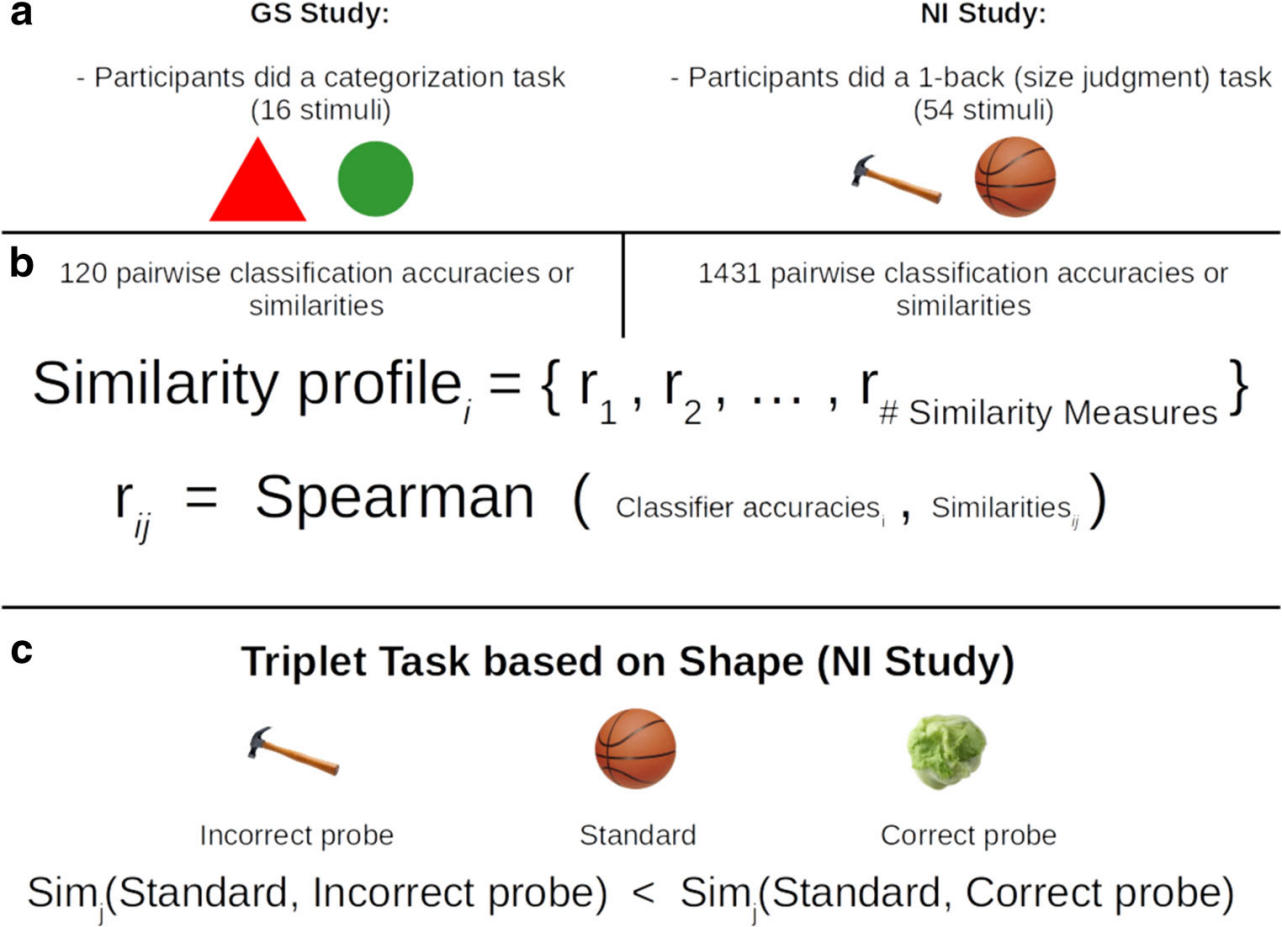
An overview of the materials and basic analyses. **a** Participants engaged either in a categorization task for the GS study or in a 1-back task for the NI study. Importantly, the tasks in the original studies are independent of the analyses we perform, which are only concerned with the fMRI activations arising from the stimulus presentations. Examples of the stimuli used for each study are shown. **b** The neural similarity analysis involved comparing *similarity* *profiles*. The similarity profile for region *i* is a vector in which each entry *j* is the Spearman correlation between similarity measure *j* and the classification accuracies of each region *i*. Each Spearman correlation involves all possible stimulus pairs (excluding an item with itself). For the similarity measure, this includes the similarity of each item with every other item. For the classifier accuracy, the accuracies of the binary classifier for the corresponding two stimulus items are included. For the GS study with 16 stimuli, each Spearman correlation involved 16 × 15/2 = 120 similarity-classifier accuracy pairs. For the NI study with 54 stimuli, each Spearman correlation involved 54 × 53/2 = 1431 similarity-classifier accuracy pairs. **c** In the triplet analysis, the question is which of the two probe items is more neurally similar to the standard using neural similarity measure *j*. When the probe that matches the standard in shape is more similar, the trial is scored as correct. All possible triplets (under a few constraints, see SI) are considered for each ROI

To showcase the distinction between similarly and classification operators, in addition to our main results, we also present results for a non-classification task that relies on neural similarity (i.e., a triplet analysis, see Fig. 3 below). In particular, we assess neural similarity between a standard stimulus and two probe stimuli, one of which matches in shape. The similarity measures that perform best (i.e., select the shape match standard) in the triplet analysis are the ones that perform best in our main decoding analyses. Critically, the stimulus classes used in the triplet analysis were not included in the decoding analysis, which highlights that similarity functions apply more broadly than classification functions and that our method for selecting the brain’s preferred similarity functions generalizes to novel stimulus classes. This result also highlights how similarity measures selected based on decoding predict performance on an independent measure (e.g., shape match) that is outside the selection procedure. Before visiting this result, we present the main results that answer key questions, such as whether the brain’s preferred similarity measures are common across regions and tasks.

## Materials and Methods

### Datasets

The analyses are based on two previous fMRI studies: a study that presented simple geometric shapes (GS) to participants (Mack et al. 2013) and a study that presented natural images (NI) to participants (Bracci and de Beeck 2016). The geometric shapes varied on four binary valued dimensions (16 stimuli total) and the natural stimuli were organized orthogonally, either by shape or by one of six categories such as fruits or tools (54 stimuli total). The GS study consisted of a visual categorization task with 20 participants and the NI study of a 1-back size judgment task with 14 participants. Further descriptions of the tasks, the stimuli, and acquisition parameters can be consulted in the SI (see Fig. 3a for an example of the stimuli for both studies). For further information, the reader should consult the source citation directly.

### Classification Analysis

Pattern classification analyses were implemented using PyMVPA (Hanke et al. 2009), Scikit-Learn (Pedregosa et al. 2011), and custom Python code. The input to the classifiers were least squares separate (LS-S) beta coefficients for each presentation of a stimulus (Mumford et al. 2012) (see SI). Basically, each beta coefficient represents the peak activation for a single presentation of a stimulus for a given voxel. Three classifiers were used for the pattern classification: Gaussian naïve Bayes, *k*-nearest neighbor, and linear support vector machine (SVM). The output of one of these classifiers was to be chosen as the best representation of the underlying similarity matrix to which all other similarity measures would be compared to (see the neural similarity analysis below). The linear SVM was implemented with the *Nu* parametrization (Schölkopf et al. 2000). This *Nu* parameter controls the fraction of data points inside the soft margin; the default value of 0.5 was used for all classifications. The *k*-nearest neighbor classifier was implemented using five neighbors. No hyperparameters required setting for the Gaussian naïve Bayes classifier.

To pick the best-performing classifier, classification was conducted on the whole-brain (no parcellation into distinct ROIs) for each study independently. All classifiers were trained with leave-one-out *k*-fold cross-validation, where *k* was equal to the number of functional runs for each participant in each study (e.g., six runs in the GS study or sixteen runs in the NI study). To do feature selection on voxels, all voxels were ordered according to their *F* values computed from an ANOVA across all class (stimuli) labels. The top 300 voxels with the highest *F* values were retained based on classifier performance (i.e., accuracy) on the test run. For these classifiers, accuracy was computed across all classes (16 classes for the GS study and 54 classes for the NI study) with a majority vote rule across all computed decision boundaries (for classifiers where this is applicable like linear SVM). This means that random classification is equal to 6.25% for the GS study and 1.85% for the NI study for this whole-brain analysis. However, for all other classification analyses, accuracy is computed as mean pairwise accuracy across all classes, which means that random classification is equal to 50%. The best-performing classifier was selected as the classifier with highest mean accuracy (mean across participants) in the GS and NI study, independently. Classifier accuracies (i.e., confusion matrices) were multiplied by negative one for the neural similarity analysis explained. This was done so that they would correlate positively with the (dis)similarity measures and facilitate presentation of results. As mentioned previously, the confusion matrices are symmetric since they are constructed from pairwise accuracies.

The following analysis was performed for each of the 110 ROIs that are described in the SI. To train the classifiers leave-one-out *k*-fold cross-validation was also used. Within each fold, a (randomly) picked validation run was used to tune the number of features (i.e., voxels) that would be selected for that fold. Thus, feature selection was done within each fold. To do this feature selection, all voxels were ordered according to their *F* values computed from an ANOVA across all class (stimuli) labels. This step aids classifier performance because it preselects task-relevant voxels (as opposed to item discriminative voxels). It is important to note that these ANOVAs were computed on the training runs but not on the validation run nor on the held-out test run, to avoid overfitting. The top *n* voxels with the highest *F* values were used to train a classifier and estimate its classification accuracy on the validation run. The number *n* that generates the highest accuracy is then chosen for the classifier and the ROI. Scipy’s *minimize_scalar* function (Jones et al. 2001) was used to optimize this validation run accuracy with respect to the top *n* voxels. After picking the top *n* voxels, the classifiers were trained on both the training runs and the validation run. Subsequently, the classifiers were tested on the held-out test run for that fold. This classification analysis was done for all possible pairwise classifications for each study (i.e., 120 pairwise classifications in the GS study and 1431 pairwise classifications in the NI study, see Fig. 3b). From this analysis, the pairwise classification accuracies were retained for both the validation run and the test run for each fold. Further ROI selection (top twelve ROIs reported in the “Results” section) is described in the SI.

### Neural Similarity Analysis

The goal of this analysis was to compare the representation of different similarity measures in the brain. The regions considered here are the ones reported in the “Results” section and described in the secondary ROI selection section in the SI. The comparison criterion was chosen as the Spearman correlation between all pairwise similarities and the classification accuracies mentioned above. This criterion was used since it avoids scaling issues. To achieve this, first all pairwise similarities (i.e., for all pairs of stimuli) were computed from the training runs defined in the classification analysis—not including the validation run. Incidentally, feature selection was also realized here. In the same fashion as in the classification analysis, all voxels were ordered according to their *F* values computed from an ANOVA across all class (stimuli) labels. Then, the top *n* voxels with the highest *F* values were retained based on the Spearman correlation of the similarities with the validation run accuracies of the classifier that were previously computed. After picking the top *n* voxels, the similarities were computed across training runs and validation run for those voxels. These similarities were then used to compute the final Spearman correlation with the classifier test run accuracies. Conducting feature selection for the similarity measures is important because different measures leverage information differently. For measures that require estimating a covariance matrix, this matrix was computed across all classes (i.e., pairwise dot product) in the training set with either Ledoit-Wolf regularization (r), diagonal regularization (d), or no regularization at all.

This analysis parallels the classification analysis in every way except that instead of optimizing model accuracy, here the optimization criterion was model correlation (i.e., Spearman correlation) with the previously computed pairwise classifier accuracies.

### Mixed-Effects Models

A mixed-effects model was performed with the lme4 package (Bates et al. 2014) for each study with Spearman correlations from the neural similarity analysis (i.e., similarity profile) as the response variable. The models contained fixed effects of similarity measure, linear SVM accuracy, participant, and ROI. Linear SVM accuracy, participant, and ROI variables only serve to account for variance and obtain better estimates. The models also contained random effects of ROI (varying per participant) and of similarity measure (varying per ROI). Model comparisons were performed between the full model and a null model without any similarity measures. Follow-up contrasts of the similarity measure were performed, compared to a baseline measure (Pearson correlation).^3^ The contrasts that compared each measure to Pearson correlation were computed with the *multcomp* package in R (Hothorn et al. 2008).

### Searchlight Analysis

This analysis allows finer spatial localization and was conducted on the union of the top 10 ROIs across both studies (see Secondary ROI selection in SI) in the native space of each subject using PyMVPA’s searchlight function. For each voxel (i.e., searchlight center), the similarity matrices were Spearman correlated with the best-performing classifier in the same fashion as in the main analysis above. For each study, the statistical maps of Euclidean and Mahalanobis(r) were compared to the statistical map of Pearson correlation, using it as a baseline measure. All maps were transformed to MNI space for this comparison. The threshold-free enhancement (TFCE)–corrected *p* values for the paired *t* statistics were computed with FSL’s randomize function with 5000 permutations. Only *t* statistics with TFCE-corrected *p* values below 0.001 were considered as significant. This conservative threshold was used to adjust for the fact that these 3 similarity measures were selected from the original 17 (i.e., *p*= 0.05/17 ≈ 0.0029).

### Triplet Analysis

As discussed in the “Introduction” section, similarity and classification are distinct concepts. To illustrate, this analysis shows how similarity measures can be used in non-classification settings involving stimuli from novel (untrained) classes. In particular, we consider a triplet analysis involving data from the NI study (Figs. 3c and 6). The analysis compared which of two probe items is more neurally similar to the standard stimulus. Trials were defined as correct when the probe that matches in shape was more neurally similar. Clusters of items organized by shape were predefined in the original NI study.

Thus, if the similarity measure was higher between standard and correct probe (which matches on shape) than it was for standard and incorrect probe (which does not match on shape), then the outcome of such a comparison was labelled with value 1, otherwise 0 (see Fig. 3c). So for each created triplet, there was always one probe that matched on shape and one that did not. All possible triplets were created with the additional constraint that the standard not be in the same nominal category as either probe; nominal categories were also predefined in the original NI study. Accuracy was computed as the number of outcomes equal to 1 divided by the number of triplets (8640 triplets total, see SI). These accuracies were correlated with the NI similarity profile explained above in the neural similarity analysis, both on the test set (which excluded the standard and correct probe on each triplet) and on the whole set of stimuli (Fig. 6).

To summarize all the methods, we first obtained the LS-S estimates of the beta coefficients, representing peak activation for a single presentation of a stimulus per voxel. Then, a black box approach was taken to pick a classifier with best decoding accuracy for each study (GS and NI) from a selection of predetermined classifiers. The union of the top 10 ROIs in decoding accuracy across both studies was used as an ROI selection criteria for further analyses. For each of these ROIs, we computed similarity profiles as a vector of Spearman correlations between pairwise classifier accuracies and pairwise similarities (for each similarity measure, see Fig. 2 and Fig. 3b). Similarity profiles were compared to each other via a mixed-effects model including contrasts with respect to a baseline measure (Pearson correlation). Finally, three measures of interest (Euclidean, Mahalanobis, and Pearson correlation) were visually inspected in the brain with a searchlight analysis. As an added bonus, we further justified the neural similarity analysis with a triplet analysis for the LS-S beta coefficients sourced from the NI study. In the triplet analysis, we compared the accuracy of each similarity measure as defined on triplets of standards, correct probes, and incorrect probes. Correctness was defined as equivalence in shape, as predetermined in the original NI study. Accuracy was defined as the proportion of standard to correct probe similarity measurements that were higher than the standard to incorrect probe similarity measurements (Fig. 3c). Accuracies from the triplet analysis were then related to the Spearman correlations from the neural similarity analysis (Fig. 6 below).

## Results

### Neural Similarity

What makes two brain states similar and does it vary across brain regions and tasks? The following analyses focus both on the performance of individual similarity measures and on the pattern of performance across a set of candidate measures, which we refer to as the *similarity profile* for an ROI (see Fig. 2).

**Fig. 4:**
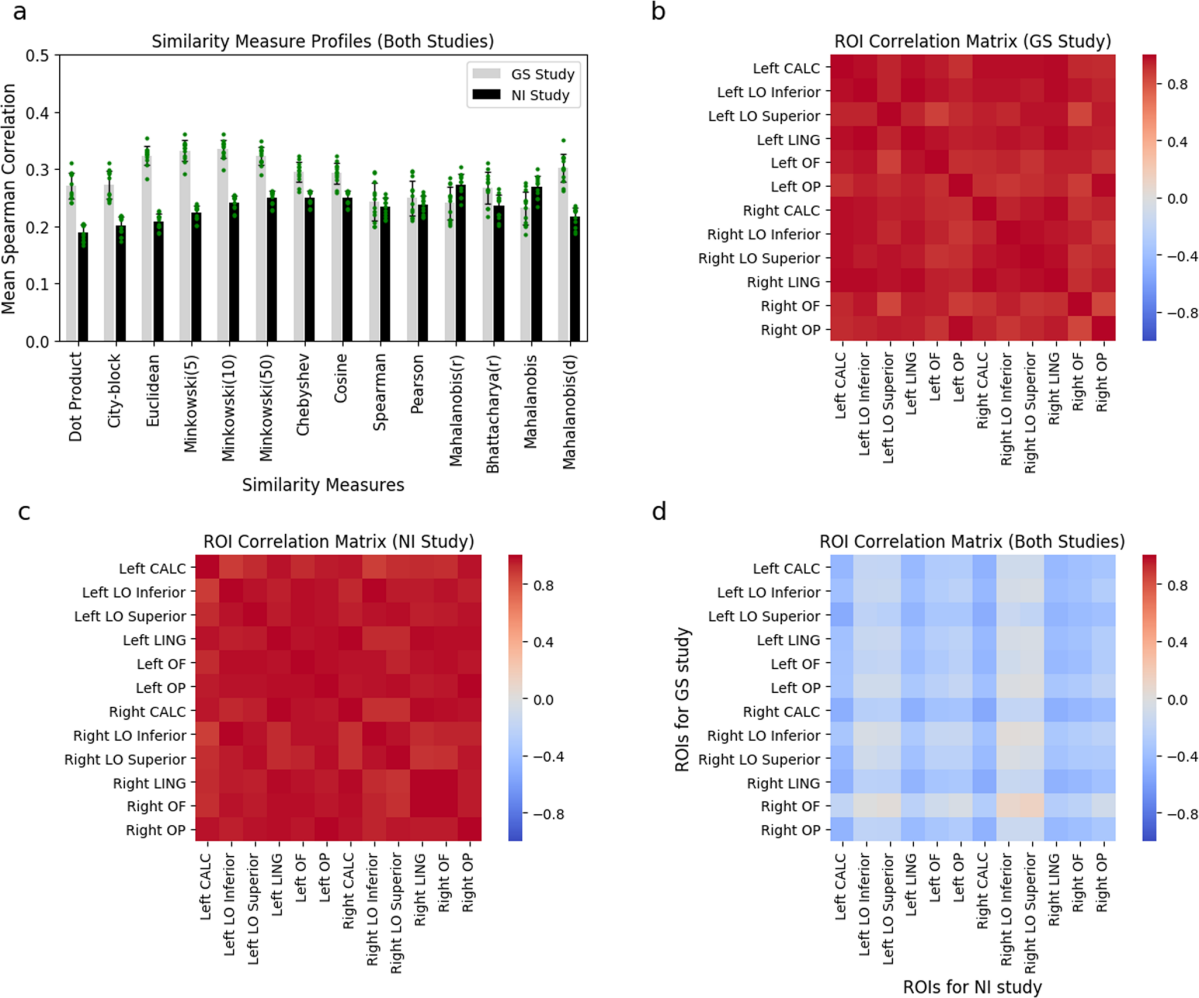
Similarity measure profiles and ROI correlation matrices. Mean Spearman correlations (**a**) for each similarity measure and the classifier confusion matrix in the GS study (grey bars) and the NI study (black bars) are displayed. To convey the variability, error bars are plotted as standard deviations, and each ROI mean is plotted as a green point. ROI correlation matrices for the GS (**b**) and NI (**c**) studies, demonstrating that the similarity profiles were alike across brain regions (i.e., were positively Pearson correlated). ROI correlation matrix (**d**) demonstrating that the similarity profiles disagreed across studies (i.e, were negatively Pearson correlated). The 12 ROIs were left and right intracalcarine cortex (CALC), left and right lateral occipital cortex (LO) inferior and superior divisions, left and right lingual gyrus (LING), left and right occipital fusiform gyrus (OF), and left and right occipital pole (OP)

As a precursor, we first tested whether similarity measures differed in their performance (Fig. 4a). Specifically, we evaluated whether certain measures better describe what makes two brain states similar by nested comparison using a mixed-effects model for each study (see “Materials and Methods”). For both studies, similarity measures differed in their performance, *χ*^2^(2) = 1720.331, *p* < 0.001; *χ*^2^(2) = 6770.249, *p* < 0.001, for the GS and NI studies, respectively.

We tested whether the similarity profile differed across brain regions within each study. The similarity profiles (i.e., mean aggregate performance across measures) were remarkably alike across ROIs (see “Materials and Methods”). High (Pearson) correlations are presented within task for both the GS study (Fig. 4b) and the NI study (Fig. 4c) between all pairs of ROIs, where mean correlation of the upper triangle is 0.95 (s.d. = 0.034) in the former and 0.96 (s.d. = 0.027) in the latter. Bartlett’s test (Bartlett 1951), which evaluates whether the matrices are different from an identity matrix, was significant for both the GS study, *χ*^2^(66) = 432.847, *p* < 0.001, and the NI study, *χ*^2^(66) = 502.7494, *p* < 0.001. Permutation tests (with 10,000 iterations), where the labels of the similarity measures were permuted, confirmed these results (*p* < 0.001). These results are consistent with the same similarity measures being used across brain regions within each study.

We tested whether similarity profiles differed between studies. The results indicated that similarity profiles differed between studies, suggesting that the operable neural similarity measures can change as a function of task or stimuli (Fig. 4d). In particular, similarity profiles between studies were negatively correlated with a mean correlation of the upper triangle of − 0.27 (s.d. = 0.148). Jennrich’s test (Jennrich 1970) showed that this matrix was different than a matrix of zeros, *χ*^2^(66) = 769.0349, *p* < 0.001. Permutation tests (10,000 iterations) with shuffling of similarity label measures also confirmed these results (*p*< 0.001).

### Searchlight Analysis

In light of these results, *post hoc* pairwise tests of each similarity against the Pearson similarity measure, which is the *de facto* default choice in the literature, were conducted. The contrasts from the mixed-effects models (mentioned above, see “Materials and Methods”) presented in Table 1 provide evidence that some similarity measures are a superior description of the brain’s similarity measure. The performance of many measures differed from Pearson, especially in the NI study. Notably, only two variants of the Mahalanobis measure and three Minkowski measures outperformed Pearson. In the GS study, we can observe that all the Minkowski distances performed better than Pearson as well as cosine, Mahalanobis(d), and the dot product. Once again, the contrasting pattern of results between the two studies is striking.

**Table 1:** Comparison of similarity measures to Pearson correlation. Top panel shows significant *z* statistics for measures worse than Pearson correlation (in brackets) and better than Pearson correlation for the GS study. Bottom panel shows the same for the NI study. *p* values are Bonferroni corrected

Similarity measure	*z*	*p*
GS study		
Minkowski(5)	12.562	< 0.001
Euclidean	12.145	< 0.001
Minkowski(10)	10.459	< 0.001
City block	10.479	< 0.001
Mahalanobis(d)	8.825	< 0.001
Minkowski(50)	6.624	< 0.001
Chebyshev	6.353	< 0.001
Cosine	4.532	< 0.001
Dot product	4.053	< 0.001
Mahalanobis	(3.161)	0.02
NI study		
Mahalanobis(r)	11.301	< 0.001
Mahalanobis	10.304	< 0.001
Minkowski(50)	4.920	< 0.001
Chebyshev	4.733	< 0.001
Minkowski(10)	4.005	< 0.001
Euclidean	(5.170)	< 0.001
Mahalanobis(d)	(7.593)	< 0.001
City-block	(10.411)	< 0.001
Cosine	(22.803)	< 0.001
Dot product	(29.547)	< 0.001

**Fig. 5:**
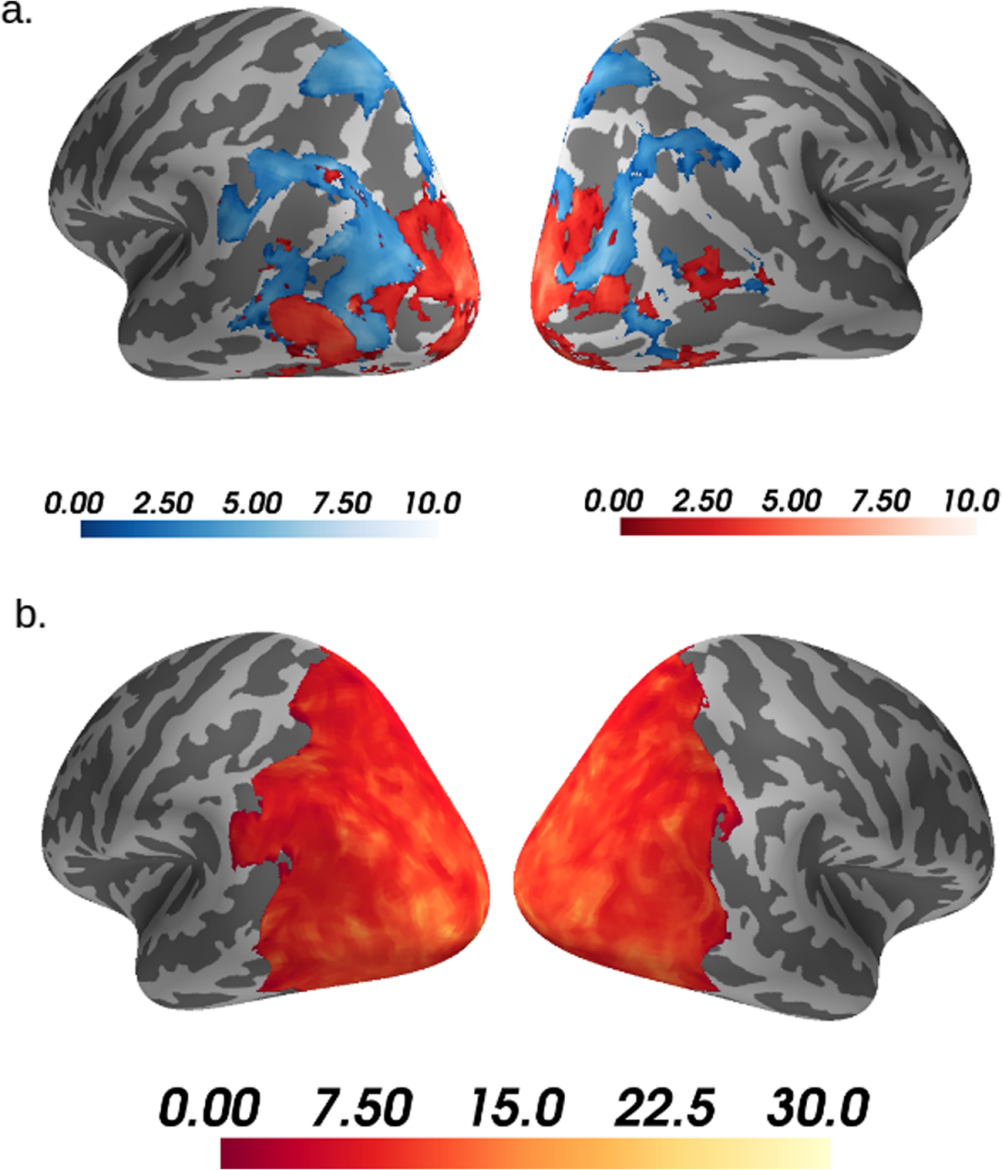
Euclidean and Mahalanobis(r) outperform Pearson. Occipito-lateral views of the left and right hemispheres for the GS study (**a**) and the NI study (**b**) displaying maximum *t* statistics where either the Euclidean measure (blue) or the Mahalanobis(r) measure (red) outperformed the Pearson correlation measure (i.e., each voxel displays the *t* statistic for the measure with highest *t*). The *t* statistics were based on a searchlight analysis of Spearman correlations of each measure with each voxel’s SVM confusion matrix (see “Materials and Methods”). Only displaying *t* statistics where *p*< 0.001 for paired sample *t* tests, TFCE corrected; computed with FSL’s randomize function with 5000 permutations, using as a mask the 12 ROIs with best accuracy (see “Materials and Methods”). Note that very few voxels only show the Euclidean measure significantly outperforming Pearson correlation in the NI study, thus do not appear in this visualization

Given the performance of the Euclidean and Mahalanobis(r) measures, and that they have been used previously in analyzing neural data (Fritsch et al. 2012; Nili et al. 2014; Persson and Rieskamp 2009; Walther et al. 2016), we selected these measures for inclusion in a searchlight analysis (Fig. 5, see “Materials and Methods” for details). By comparing the Euclidean and Mahalanobis(r) measures to Pearson correlation on a voxel-by-voxel basis for the 12 ROIs, we aimed to provide a visualization of the performance of similarity measures across regions and studies. Figure 5 illustrates the regions where these two measures outperform Pearson correlation, displaying the maximum *t* for voxels where both Euclidean and Mahalanobis overlap (see SI for visualizations of the overlap).

In the NI study, the Mahalanobis(r) measure dominated (Fig. 5b), confirming the results from the previous analyses. In contrast, in the GS study (Fig. 5a), Euclidean dominates in some regions whereas Mahalanobis(r) dominates in others. Despite it being a *de facto* standard, Pearson similarity was never the top measure. For this *post hoc* analysis, the measures were compared using permuted paired sample *t* statistics for each voxel. Positive *t* statistics that survived threshold-free cluster enhancement (TFCE) correction with *p*< 0.001 are presented in Fig. 5 (see “Materials and Methods” for the rationale behind this threshold).

### Triplet Analysis

In this section, we show that neural measures that perform best in our decoding analysis perform best in the triplet analysis, despite the entire classes used in the triplet analysis being withheld from the decoding analysis. These results indicate that approximating the information available in a brain state through decoding can select similarity measures that broadly generalize and perform sensibly in novel tasks.

**Fig. 6:**
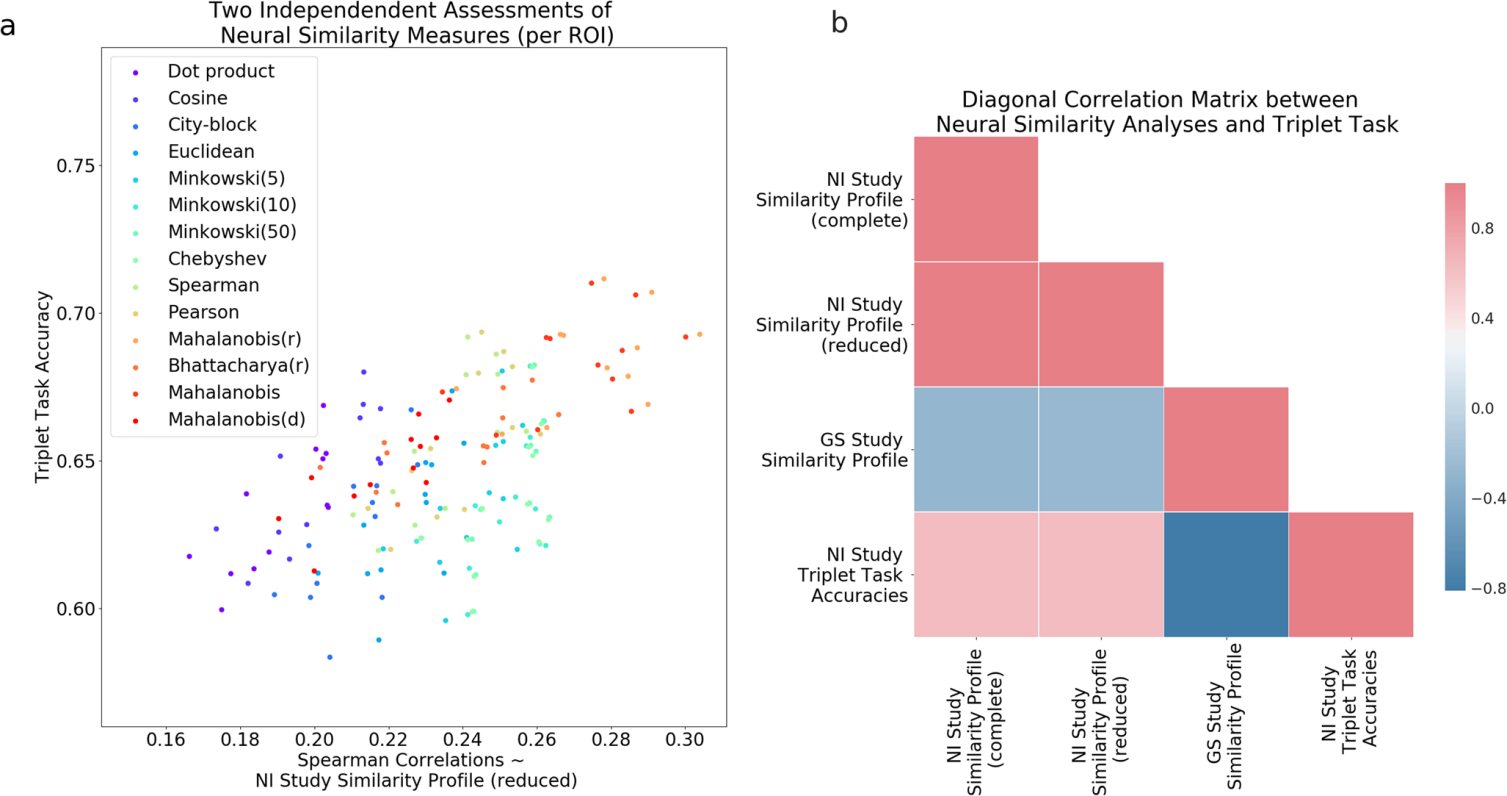
Triplet analysis accuracies correlate with NI study similarity profiles. In **a**, each data point represents one similarity measure per region of interest. The Spearman correlations in **a** have been recalculated with the removal of held-out pairs used in the triplet analysis (where each pair is the standard and the correct probe), thus termed NI study similarity profile (reduced). In **b**, we Pearson correlate the similarity profiles from the neural similarity analysis with the accuracies derived from the triplet analysis as well as with each other. NI study similarity profile (complete) and GS study similarity profile are the same Spearman correlations as displayed in Fig. 4a

The triplet analysis allows a separate evaluation of the similarity measures of interest by comparing the accuracies in such a task to the similarity profile of the NI study (Fig. 6a); Pearson correlation of *r*(12) = 0.63,*p* = 0.017, across the fourteen similarity measures of interest. For this association, the scatterplot in Fig. 6a shows the variance associated to the twelve regions of interest presented above. Measures like Mahalanobis and Mahalanobis(r) clearly do best; in line with the original similarity profile of the NI study reported in the neural similarity analysis (Fig. 4a). The similarity profile correlations were adjusted to account for the held-out pairs from the triplet analysis (with standard and correct probe removed), thus termed (*reduced*) in contrast to the original profile and reported here as (*complete*) (see “Materials and Methods”). In Fig. 6b, all the similarity profiles are related amongst each other and with the triplet analysis accuracies. Most notably, the bottom row of the diagonal matrix displays how the triplet analysis accuracies also Pearson correlate negatively with the GS study similarity profile as in Fig. 4d, *r*(12) = − 0.81,*p* < 0.001. For comparison purposes, we also present the Pearson correlation of the triplet analysis accuracies with NI study similarity profile (complete), *r*(12) = 0.63,*p* = 0.016. The triplet analysis is thus an independent assessment of the validity of our neural similarity analysis.

These results clearly demonstrate independence from our method of selecting similarity measures based on a decoding approach that approximates the information available in a brain state. In the triplet analysis, similarity measures that performed best in our neural similarity analysis also performed best in this novel task involving untrained classes. More supporting evidence distinguishing classification from similarity is also presented in the SI; the best-performing classifier is a linear SVM for both the GS and NI study, whereas we find differences in similarity profiles between studies. Clearly, similarity is not a simple recapitalization of classification.

## Discussion

One fundamental question for neuroscience is what makes two brain states similar. This question is so basic that in some ways it has been overlooked or sidestepped by assuming that Pearson correlation captures neural similarity. Here, we made an initial effort to evaluate empirically which of several competing similarity measures is the best description of neural similarity.

Our basic approach was to characterize the question as a model selection problem in which each similarity measure is a competing model. The various similarity measures (i.e., models) competed to best account for the data, which was the confusion matrix from a classifier (i.e., decoder) that approximated the information present in a brain region of interest. The motivation for this approach is that more similar items (e.g., a sparrow and a robin) should be more confusable than dissimilar items (e.g., a sparrow and a moped). Thus, the test of a similarity measure, which is a pairwise operator on two neural representations, is how well its predicted neural similarities agree with the classifier’s confusion matrix.

At this early juncture, basic questions, such as whether different brain regions use different measures of similarity and whether the nature of neural similarity is constant across studies remained unanswered. Our results indicated that the neural similarity profile (i.e., the pattern of performance across candidate similarity measures) was constant across brain regions within a study, though strongly differed across the two studies we considered. Furthermore, Pearson correlation, the *de facto* standard for neural similarity, was bested by competing similarity measures in both studies.

Support for the validity of our method came from the follow-on triplet analysis in which we tested the ability of the similarity measures to select which of two probe items was most neurally similar to a comparison item. Similarity measures that performed best at this task (by selecting the probe that matched the comparison in stimulus shape) were those that also performed best under our decoding approach to evaluating neural similarity, despite the fact that the stimuli and classes used in the triplet were withheld from the decoding analyses. These results establish that our method of evaluating similarity measures selects measures that generalize well to novel tasks and stimulus classes. It also highlights that similarity and classification are distinct functions.

Accordingly, we report results in the SI in which the best-performing similarity measures vary while the best-performing classifier remains constant, providing an illustration of how similarity and classifier performance can diverge. Of course, despite similarity and classification being distinct, the classifier used to estimate the information present in a brain region could bias the results; although, it is not clear if this can be proven in the general case. For example, a case could be made for the dot product—as an internal operation of the linear SVM—to be biased in picking Pearson correlation since they both attend to vector directions, which we do not observe in our results. Indeed, our method is classifier agnostic, selecting the classifier that extracts the most information from a given brain state. If there should be some general formal relation between similarity functions and classifiers, this would only improve the theoretical interpretation of the brain’s similarity measure without compromising their status as different concepts. We recommend the procedure we followed: consider a variety of classifiers and choose the best-performing classifier independently of how the neural similarity measures perform (see SI). In practice, this means that an advance in classifier techniques would invite reconsidering how neural similarity measures perform.

One question is why the neural similarity profile would differ across studies. There are host of possibilities. One is that the nature of stimuli drove the differences. The stimuli in the GS study were designed to be psychologically separable, consisting of four independent binary dimensions (color: red or green, shape: circle or triangle, size: large or small, and position: right or left). These stimuli were designed to conform to a Euclidean space so that cognitive models assuming such similarity spaces could be fit to the behavioral data. Accordingly, in our analyses, the neural similarity measures from the Minkowski family (including Euclidean) performed best. In contrast, the NI study consisted of naturalistic stimuli (photographs) that covaried in a manner not easily decomposable into a small set of shared features. One possibility is that these types of complex feature distributions are better paired with the Mahalanobis measure (cf. Diedrichsen and Kriegeskorte (2017)). Of course, task also varied with stimuli which offers yet another possible higher-level explanation for the differences observed in neural similarity performance. For example, the task in the GS study emphasized analytically decomposing stimuli into separable dimensions whereas holistic processing of differences was a viable strategy in the NI study. In general, different tasks will require neural representations that differ in their dimensionality or complexity (Ahlheim and Love 2018), which has ramifications for what similarity measure is most suitable.

A host of other concerns related to data quality may also influence how similarity measures perform. The nature of fMRI BOLD response itself places strong constraints on the types of models that can succeed (Guest and Love 2017), which suggests that future work should apply the techniques presented here to other measures of neural activity. Regardless of the measure of neural activity, more complex models of neural similarity will require higher quality data to be properly estimated. For example, measures such as Mahalanobis or Bhattacharyya need to estimate inverse covariance matrices. These matrices grow with the square of the number of vector components which approaches both numerical and statistical unreliability when the number of components approaches the number of observations. For these reasons, we optimized the number of top features (i.e., voxels) separately for each similarity measure (see “Materials and Methods”), except in the searchlight analysis where this was not possible. We also considered regularized versions of similarity measures, such as Mahalanobis(d), that should be more competitive when data quality is limited.

Although the similarity measures considered are relatively simple, they make a host of assumptions that are theoretically and practically consequential. For example, angle measures, such as Pearson correlation, are unconcerned with differences in the overall level of neural activity, an assumption that strongly contrasts with magnitude measures, such as those in the Minkowski family (e.g., Euclidean measure). Therefore, the choice of similarity measure is central to any mechanistic theory of brain function and has practical ramifications when analyzing neural data, such as when characterizing neural representation spaces. In this light, operations that may seem routine, such as normalizing data in various ways, can affect the interpretation of results. For example, vector cosine only differs from dot product by virtue of normalizing by the magnitude of the two state vectors.

As mentioned previously, the space of possible similarity measures is uncountably infinite and new measures routinely enter the literature (Allefeld and Haynes 2014; Walther et al. 2016). Such studies may focus more on reliability criteria, rather than on modelling the informational content of a brain state (Walther et al. 2016). The distinction is subtle but it does describe the difference between assessing a similarity measure as a model of neural and cognitive phenomena as opposed to assessing a measure’s suitability for a data analysis pipeline. In line with our main results, sometimes new measures like crossnobis perform well, and sometimes they fail (Charest et al. 2018). Here, we aimed to include representative measures from the main families of similarity measures we identified (see Fig. 1, left side). Others are free to replicate our analyses with alternative sets of measures. For example, a different approach entirely could be to learn the metric directly from the data, but perhaps limiting its theoretical interpretability (Xing et al. 2003).

Although we focus on the BOLD response, our approach applies equally to other neural measures, such as single-unit recordings, perhaps enabling the study of non-smooth similarity measures. One important open question is whether the same similarity measures perform well across measures that differ dramatically in terms of spatial and temporal resolution, as well as the aspects of neural activity they capture. Likewise, our approach can be applied to complex artificial neural networks, such as deep convolutional neural networks (CNN), which have become popular in neuroscience by virtue of their ability to track neural activity along the ventral stream during object recognition tasks (Yamins and DiCarlo 2016). In standard neural networks, the basic mathematics of integrate-and-fire artificial neurons (i.e., units) can be viewed as a similarity operation, namely a dot product between the weight representation of the unit and the activity pattern at the previous layer. Alternatively, many of the other similarity functions we considered are differentiable and could be used in CNNs trained through backpropagation to perhaps provide better performance and agreement with neural measures. The question of which similarity functions manifest at the unit level of a CNN vs. at a larger organizational level recapitulate the previous discussion of the human brain. Other future directions include evaluating different similarity kernels within classification procedures, as an alternative to assessing neural similarity computations. The work presented here adds to the general effort of constraining cognitive models with neural data (Turner et al. 2018). Neural similarity measures, as inferred here, could be used as building blocks for cognitive models, though the mapping from model components to voxels could be quite complex.

In conclusion, we took a step toward determining what makes two brain states similar. Working with two fMRI datasets, we found that the best-performing similarity measures are common across brain regions within a study, but vary across studies. Furthermore, we found that the *de facto* similarity measure, Pearson correlation, was bested in both studies. Although follow-up work is needed, the current findings and technique suggest a host of productive questions and have practical ramifications, such as determining the appropriate measure of similarity before conducting a neural representational analysis. In time, efforts making use of this and similar approaches may lead to mechanistic theories that bridge neural circuits, related measurement data, and higher-level descriptions.

## Electronic supplementary material

Below is the link to the electronic supplementary material.

(PDF 300 KB)

(TEX 28.9 KB)

(PNG 120 KB)

## Footnotes

1 Anisotropic measures should not be confused with asymmetric measures; the latter gives different values based on which stimulus is measured first (Nosofsky 1992; Tversky 1977).

2 The matrix is symmetric since it is constructed with pairwise accuracies (see “Materials and Methods”).

3 A full model that included both studies was not possible due to convergence issues.
