# Supplementary material for: Measures of Neural Similarity
Source: Comput Brain Behav. 2019 Dec 2;3(4):369–83. doi: 10.1007/s42113-019-00068-5 (PMC7671987; doi:10.1007/s42113-019-00068-5)
Supplement: Supplementary file 1 — (PDF 300 KB) [file 42113_2019_68_MOESM1_ESM.pdf]

Supplementary Information

A. Task descriptions and fMRI parameters

A.1 Geometric shapes (GS) study

The GS study (Mack et al., 2013) presented sixteen objects in total, which varied on four different binary features: (color: red or green, shape: circle or triangle, size: large or small, and position: right or left). Participants in this study were trained to do a categorization task. They were first trained on five objects of one category and four of the other (nine objects total during training) with twenty repetitions of each object. During the anatomical scan, participants saw four more repetitions of the training items as a refresher. Then during the functional scanning phase, participants were asked to categorize the nine familiar objects they saw during the training phase and seven novel objects they had not seen before. Each trial during the functional scanning phase lasted 10 seconds; 3.5 seconds where one of the sixteen objects (nine training stimuli and seven novel transfer stimuli) was presented after which a fixation cross was presented for 6.5 seconds. No feedback was provided during this phase. Each stimulus was presented three times within a run across six runs resulting in each stimulus being presented a total of eighteen times during the functional scanning phase except for one participant who only participated in five runs of the scanning phase.

Whole-brain imaging data were acquired on a 3.0T GE Sigma MRI system (GE Medical Systems). Structural images were acquired using a T2-weighted flow-compensated spin-echo pulse sequence (TR=3s; TE=68ms, 256x256 matrix, 1x1mm in-plane resolution) with thirty-three 3-mm thick oblique axial slices (0.6mm gap), approximately 20 off the AC-PC line. Functional images were acquired with an echo planar imaging sequence using the same slice prescription as the structural images (TR=2s, TE=30.5ms, flip angle=73, 64x64 matrix, 3.75x3.75 in-plane resolution, bottom-up interleaved acquisition, 0.6mm gap). An additional high-resolution T1-weighted 3D SPGR structural volume (256x256x172 matrix, 1x1x1.3mm voxels) was acquired for registration and cortex parcellation.

A.2 Natural images (NI) study

The NI study (Bracci and de Beeck, 2016) presented fifty-four objects in total, which varied in two ways. The 54 stimulus items were conceived to either be organized by category (6 categories: minerals, animals, fruits/vegetables, music, sports, or tools) or by their silhouette (9 silhouettes) which cut orthogonally across the category distinction. Participants in this study were asked to perform a 1-back real-world size judgment task (i.e., to respond according to whether the object on the previous trial was larger or smaller than the current image on screen). Participants were scanned on two separate sessions (different days). Each session consisted of eight functional scanning runs resulting in sixteen runs total except for one participant for which four of the runs of the first session were lost due to scanning issues. Each one of the fifty-four objects were presented twice within each run in a randomized sequence. This resulted in each object being presented a total of thirty-two times (or twenty-four times for the participant that only had twelve runs). On each trial, each object was presented for 1.5 seconds after which a fixation cross was presented for 1.5 seconds. Each run started with a fixation cross for fourteen seconds and ended with a fixation cross for fourteen seconds. Thirty-six fixation trials lasting three seconds each were also randomly presented within each run.

Data collection was performed on a 3T Philips scanner with a 32-channel coil at the Department of Radiology of the University Hospitals Leuven. MRI volumes were collected using echo planar (EPI) T2*-weighted scans. Acquisition parameters were as follows: repetition time (TR) of 2 s, echo time (TE) of 30 ms, flip angle (FA) of 90, field of view (FoV) of 216 mm, and matrix size of 72x72. Each volume comprised 37 axial slices (covering the whole brain) with 3 mm thickness and no gap. The T1-weighted anatomical images were acquired with an MP-RAGE sequence, with 1x1x1 mm resolution.

A.3 fMRI preprocessing

The original raw (NIfTI formatted) files from both studies were preprocessed and analyzed using FSL 4.1 (Jenkinson et al., 2012). Functional images were realigned to the first volume in the time series to correct for motion, co-registered to the T2-weighted structural volume, high-pass filtered (128s), and detrended to remove linear trends within each run. All analyses were performed in the native space of each participant.

A.4 Trial-by-trial estimates

For both studies, after preprocessing the fMRI data with FSL, the method suggested by Mumford et al. (2012) known as LS-S (least squares separate) beta estimation was used to get a coefficient estimate for each individual presentation of each object. This method consists of calculating a general linear model for each object presentation with only two regressors; one regressor representing the effect of interest (the object presentation in question) and another regressor representing all other object presentations within the respective run. This procedure was done for each run separately to preserve as much statistical independence as possible between runs. Such a step is necessary for doing the multivoxel pattern analysis. After successfully estimating the object presentation coefficients within each run, these were then concatenated into a single 4D NIfTI formatted file. Furthermore, all runs were subsequently aligned to the last run within each study (e.g. the sixth run in the GS study or the sixteenth run in the NI study). The runs were then concatenated into a single 4D NIfTI formatted file for each participant within each study.

B. Regions of interest from the Harvard-Oxford atlas

B.1 Initial region of interest (ROI) selection

The Harvard-Oxford cortical and subcortical structural atlases provided with FSL (Jenkinson et al., 2012) were used to parcellate the different anatomical regions for each participant. A total of 110 regions of interest were used as masks that would be used in the multivoxel pattern analyses. The goal was to evaluate classifier accuracy across the whole brain (except for areas like cerebral white matter or the lateral ventricles). More areas could have been excluded based on a priori hypotheses of where similarity signals would arise. However, including areas where no signal was expected served as an informal control for the method and still retained the possibility that similarity signals could have been found in otherwise unexpected brain regions. The masks were transformed from MNI space to each participants native space. This masking by anatomical region can be considered the first part of a feature selection procedure. Feature selection was also done within each region of interest for each participant (see Materials and Methods). All regions from the Harvard-Oxford atlas were included in the analyses except for cerebral white matter, the lateral ventricles, left and right cerebral cortex, and the brain stem. This results in 48 cortical regions and 7 subcortical regions; doubling for lateralization results in the 110 regions of interest.

B.2 Cortical regions of interest

Frontal Pole, Insular Cortex, Superior Frontal Gyrus, Middle Frontal Gyrus, Inferior Frontal Gyrus (pars triangularis), Inferior Frontal Gyrus (pars opercularis), Precentral Gyrus, Temporal Pole, Superior Temporal Gyrus (anterior division), Superior Temporal Gyrus (posterior division), Middle Temporal Gyrus (anterior division), Middle Temporal Gyrus (posterior division), Middle Temporal Gyrus (temporooccipital part), Inferior Temporal Gyrus (anterior division), Inferior Temporal Gyrus (posterior division), Inferior Temporal Gyrus (temporooccipital part), Postcentral Gyrus, Superior Parietal Lobule, Supramarginal Gyrus (anterior division), Supramarginal Gyrus (posterior division), Angular Gyrus, Lateral Occipital Cortex (superior division), Lateral Occipital Cortex (inferior division), Intracalcarine Cortex, Frontal Medial Cortex, Juxtapositional Lobule Cortex (formerly Supplementary Motor Cortex), Subcallosal Cortex, Paracingulate Gyrus, Cingulate Gyrus (anterior division), Cingulate Gyrus (posterior division), Precuneous Cortex, Cuneal Cortex, Frontal Orbital Cortex, Parahippocampal Gyrus (anterior division), Parahippocampal Gyrus (posterior division), Lingual Gyrus, Temporal Fusiform Cortex (anterior division), Temporal Fusiform Cortex (posterior division), Temporal Occipital Fusiform Cortex, Occipital Fusiform Gyrus, Frontal Operculum Cortex, Central Opercular Cortex, Parietal Operculum Cortex, Planum Polare, Heschl's Gyrus (includes H1 and H2), Planum Temporale, Supracalcarine Cortex, & Occipital Pole.

B.3 Subcortical regions of interest

Thalamus, Caudate, Putamen, Pallidum, Hippocampus, Amygdala, & Accumbens.

B.4 Secondary ROI selection

The 110 ROIs were rank ordered by mean classifier accuracy (mean across participants) within each study. Subsequently, the union of the top ten ROIs was selected for the neural similarity analysis. This procedure was done to ensure that the ROIs used to evaluate the similarity measures was based on brain areas with adequate signal-to-noise ratio. The 12 ROIs as reported in the Results were left and right intracalcarine cortex (CALC), left and right lateral occipital cortex (LO) inferior and superior divisions, left and right lingual gyrus (LING), left and right occipital fusiform gyrus (OF), and left and right occipital pole (OP).

C. Classifier selection

The best performing classifier was chosen out of three candidates; Gaussian naïve Bayes (GNB), k-nearest neighbor (KNN), and linear support vector machine (SVM). These classifiers were chosen because they are commonly used in data analysis, both inside and outside the field of neuroimaging, and they compute classification in very distinct ways (see Pereira et al., 2009).

The linear SVM classifier was the clear winner across both studies, thus was chosen as our gold standard approximation to the brain's similarity measure. The performance of the linear SVM classifier compared to the other two classifiers is shown in Table C1.

	GS Study		NI study	
	**mean**	**s.d.**	**mean**	**s.d.**
Linear SVM	20.49%	12.64%	23.51%	5.50%
GNB	15.00%	8.79%	10.24%	2.84%
KNN	14.51%	8.50%	8.49%	3.09%
Random classification		6.25%		1.85%
	t	p	t	p
Linear SVM vs. GNB	5.22	< 0.001	14.33	< 0.001
Linear SVM vs. KNN	4.59	< 0.001	17.80	< 0.001
degrees of freedom		19		13

Table C1. Linear SVM is best-performing classifier in both studies. Top panel shows mean accuracy and standard deviations (s.d.) (across participants) for each classifier. Bottom panel shows t-tests comparing the best-performing classifier (linear SVM) to the other two classifiers.

In addition to comparing the performance of the classifiers judged by their performance accuracy, the confusion matrices between classifiers - from the same analysis - were also compared. Although the classifiers are quite distinct algorithmically speaking, extreme differences between their confusion matrices would be unlikely. Indeed it was the case that the average correlations (averaged across subjects) were all significantly above zero for both studies. In the GS study, linear SVM correlated highest with GNB (m = 0.47, s.d. = 0.172, $t(19) = 12.01$, $p < 0.001$), second highest with KNN (m = 0.37, s.d. = 0.197, $t(19) = 8.21$, $p < 0.001$), and GNB correlated with KNN in third place (m = 0.32, s.d. = 0.195, $t(19) = 7.06$, $p < 0.001$).

In the NI study, linear SVM correlated highest with GNB (m = 0.35, s.d. = 0.072, $t(13) = 17.55$, $p < 0.001$), second highest with KNN (m = 0.29, s.d. = 0.080, $t(13) = 13.06$, $p < 0.001$), and GNB correlated with KNN in third place (m = 0.22, s.d. = 0.091, $t(13) = 8.94$, $p < 0.001$). These results provide supplementary support for choosing linear SVM as the brain's gold standard for these two datasets given that it's confusion matrix correlates highest with the confusion matrices of the other two classifiers.

Thus, the linear SVM classifier was optimized for each of the initial 110 ROIs. The ROIs were rank-ordered in terms of accuracy in each study and the union of the top 10 ROIs across both studies was: left and right intracalcarine cortex (CALC), left and right lateral occipital cortex (LO) inferior division, left and right lateral occipital cortex (LO) superior division, left and right lingual gyrus (LING), left and right occipital fusiform gyrus (OF), and left and right occipital pole (OP). This resulted in a secondary ROI selection of 12 ROIs with best (linear SVM) classifier accuracy.

Classifications were performed pairwise for this analysis and thus random classification was expected at 50% for both studies (see Materials and Methods). The mean accuracy for the linear SVM classifier in the 12 regions of interest was 59.47% (s.d. = 7.97%) in the GS study and 78.43% (s.d. = 7.41%) in the NI study. The best-performing classifier (linear SVM) was performing above 50% chance level in both studies; $t(19) = 5.18$, $p < 0.001$, in the GS study and $t(13) = 13.84$, $p < 0.001$, in the NI study (degrees of freedom are based on number of participants for each study). This provides reassurance that the ROIs that were selected indeed have information regarding stimuli presentation. Classification accuracy for the NI study was higher than in the GS study $t(32) = 6.82$, $p < 0.001$, showing a potential difference in data quality due to the higher number of observations per stimuli in the NI study (see Materials and Methods).

D. Triplet analysis

The original NI study predefined 9 shape groupings and 6 nominal categories (fruits/veg, animals, minerals, music, sports, and tools). Shape and nominal category were orthogonal to each other. For each shape grouping, there were 30 pairs where one stimulus was a standard and another serving the role of a correct probe. Thus, there were 135 pairs of stimuli (across the 9 shape groupings) that matched on shape but doubled to 270 pairs to account for the role of standard or correct probe that each stimuli could take, respectively. For each possible pairing of standard and correct probe, there

were 32 possible incorrect probes; after accounting for the constraint that the incorrect probe not match in shape or nominal category to either the standard or the correct probe. This means the number of possible triplets was 8640 (i.e., 270 valid pairs times 32 possible incorrect probes).

E. Similarity measures

The following similarity measures were evaluated: dot product, cosine distance, city-block (Manhattan), Euclidean, three variants of Minkowski (with norms 5, 10 and 50), Chebyshev, Spearman correlation, Pearson correlation, three variants of Mahalanobis, three variants of Bhattacharyya, variation of information, and distance correlation. City-block, Euclidean, Minkowski, Chebyshev, Mahalanobis, Bhattacharyya and variation of information are proper distance metrics; to convert them to similarity measures they were multiplied by minus one. Other linking functions between similarities and distances are possible, as in a negative exponential (Shepard, 1987), but not relevant here since our optimization criterion was Spearman correlation. The three variants of Mahalanobis and Bhattacharyya were due to the way the sample covariance matrix was regularized; either no regularization, Ledoit-Wolf shrinkage (implemented through Scikit-Learn, Ledoit and Wolf, 2004; Pedregosa et al., 2011) or diagonal regularization. Diagonal regularization was defined as the sample covariance matrix with all the off-diagonal elements set to zero (see below); such as measure is also known as the normed Euclidean distance. Note that city-block, Euclidean, and Chebyshev are also special cases of the Minkowski measure where the norms are set to one, two and infinity, respectively. To keep calculations consistent across all similarity measures, vector representations for each stimulus were defined as the mean vectors across trial presentations for that stimulus. Below are the equations for each similarity measure and the covariance matrix regularization procedures.

In constructing the similarity profiles, we only used similarity measures that presented a mean Spearman correlation within three median absolute deviations away from the group average (group refers to measures here). Measures that did not meet these criteria were considered outliers (these measures were close to zero mean Spearman correlation). The median Spearman correlation across the 18 similarity measures evaluated was 0.203 for the GS study 0.125 and for the NI study and their median absolute deviation was 0.0482 for the GS study and 0.0234 for the NI study. The mean Spearman

correlations (across participants) and the standard deviations for the measures that were more than three median absolute deviations away from the group average were: Bhattacharya without covariance matrix regularization (mean = 0.001 and s.d. = 0.004 for the GS study, mean = 0.0002 and s.d. = 0.0006 for the NI study), Bhattacharya (d) (with diagonal regularization) (mean = -0.0005 and s.d. = 0.003 for the GS study, mean = -0.0001 and s.d. = 0.0007 for the NI study), variance of information (mean = -0.04 and s.d. = 0.037 for the GS study, mean = -0.012 and s.d. = 0.004 for the NI study), and distance correlation (mean = -0.037 and s.d. = 0.026 for the GS study, mean = -0.0009 and s.d. = 0.0038 for the NI study). These statistics were computed across the 110 original ROIs.

Below is a list of the equations for each measure considered.

For two classes represented as vectors

$$X = (x_1, x_2, ..., x_n) \in \mathbb{R}^n$$

and

$$Y = (y_1, y_2, ..., y_n) \in \mathbb{R}^n$$

where each component is computed as the arithmetic mean across m observations (trial-by-trial β coefficients) per class, per run, and n is the number of voxels. This notation is valid except for where these vectors show subscripts denoting individual observations as opposed to mean vectors (this is only the case when discussing distance correlation).

Dot product

$$XY^T$$

Cosine distance

The (negative) cosine distance is:

$$-(1 - \frac{XY^T}{\|X\|_2 \|Y\|_2})$$

where $\|\cdot\|_2$ denotes the L2 (Euclidean) norm.

Minkowski distance

The (negative) Minkowski distance is:

$$-\left(\sum_{i=1}^{n}|x_i - y_i|^p\right)^{1/p}$$

For the city-block distance $p = 1$, for the Euclidean distance $p = 2$, and for the Chebyshev distance $p = \infty$.

Pearson correlation

$$\frac{\sum_{i=1}^{n}(x_i - \bar{x})(y_i - \bar{y})}{\sqrt{\sum_{i=1}^{n}(x_i - \bar{x})^2}\sqrt{\sum_{i=1}^{n}(y_i - \bar{y})^2}}$$

where $\bar{x}$ and $\bar{y}$ are the component-wise arithmetic means of vectors X and Y, respectively.

Spearman correlation

$$1 - \frac{6\sum_{i=1}^{n}(rg(x_i) - rg(y_i))^2}{n(n^2 - 1)}$$

where $rg(x_i)$ and $rg(y_i)$ are the ranks of the values x_i and y_i, respectively. This formulation assumes distinct integer rankings.

Mahalanobis distance

The (negative) Mahalanobis measure between two random vectors coming from the same multivariate normal distribution is:

$$-\sqrt{(X - Y)^T\Sigma^{-1}(X - Y)}$$

where Σ is the $n \times n$ covariance matrix between voxels.

Bhattacharyya distance

The (negative) Bhattacharyya measure between two multivariate normal distributions $\mathcal{N}(X, \Sigma_X)$ and $\mathcal{N}(Y, \Sigma_Y)$, where each voxel-by-voxel covariance matrix Σ_X and Σ_Y is estimated separately for each class X and Y, respectively, is:

$$-\left(\frac{1}{8}(X - Y)^T\bar{\Sigma}^{-1}(X - Y) + \frac{1}{2}ln\left(\frac{det\bar{\Sigma}}{\sqrt{det\Sigma_X det\Sigma_Y}}\right)\right)$$

where

$$\bar{\Sigma} = \frac{\Sigma_X + \Sigma_Y}{2}$$

Distance correlation

The distance correlation is equal to 1 when X and Y span the same linear subspace under some linear transformation and 0 when X and Y are independent. It is defined as:

$$\frac{dCov(X,Y)}{\sqrt{dVar(X)dVar(Y)}}$$

where $dCov^2(X,Y)$ is

$$\frac{1}{m^2}\sum_{j=1}^{m}\sum_{k=1}^{m} A_{j,k}B_{j,k}$$

and $dVar^2(X)$ is

$$\frac{1}{m^2}\sum_{j=1}^{m}\sum_{k=1}^{m} A_{j,k}^2$$

where $A_{j,k}$ is the matrix computed from doubly-centering the matrix $a_{j,k}$ (subtracting row and column means while adding the grand mean), where

$$a_{j,k} = ||X_j - X_k||_2$$

Thus, $B_{j,k}$ is computed from $b_{j,k}$, where

$$b_{j,k} = ||Y_j - Y_k||_2$$

These pairwise distance matrices are computed from distances between observations.

Variation of information

For two classes X and Y represented as two multivariate Gaussian distributions, the (negative) Variation of information is

$$VI(X;Y) = I(X;Y) - H(X,Y)$$

where $H(X)$ is the entropy of X and $I(X;Y)$ is the mutual information between X and Y.

For a multivariate Gaussian X, $H(X)$ is:

$$\frac{1}{2}ln(det(2\pi e\Sigma_X)) * n$$

where n is the number of observations. The mutual information between X and Y is:

$$\frac{1}{2}ln(\frac{det\Sigma_X det\Sigma_Y}{det\Sigma^*})$$

where Σ^*

$$= \begin{bmatrix} \Sigma_X & \Sigma_{XY} \\ \Sigma_{YX} & \Sigma_Y \end{bmatrix}$$

and Σ_{XY} is the between-class voxel covariance matrix. Σ_{YX} is the transpose of Σ_{XY}. Σ_X and Σ_Y are the same voxel-by-voxel covariance matrices used in computing the Bhattacharyya distance.

Covariance matrix regularization

Two types of covariance matrix regularization were used for the Mahalanobis distance: diagonal regularization and Ledoit-Wolf regularization.

Diagonal regularization

Diagonal regularization for a covariance matrix Σ was computed as $\Sigma \circ I$, where $\circ$ is the hadamard product (element-wise multiplication) and I is the identity matrix.

The distance measure that comes as a result of this type of regularization, when applied to the covariance matrix of the Mahalanobis distance, is also known as the normed Euclidean distance.

Ledoit-Wolf regularization

Ledoit-Wolf regularization for a covariance matrix Σ was computed as:

$$(1 - shrinkage)\Sigma + (shrinkage)(\mu)I$$

where $\mu = trace(\Sigma)/n$ and the optimal shrinkage parameter is a value between 0 and 1 estimated according to the derivation in (Ledoit and Wolf, 2004).

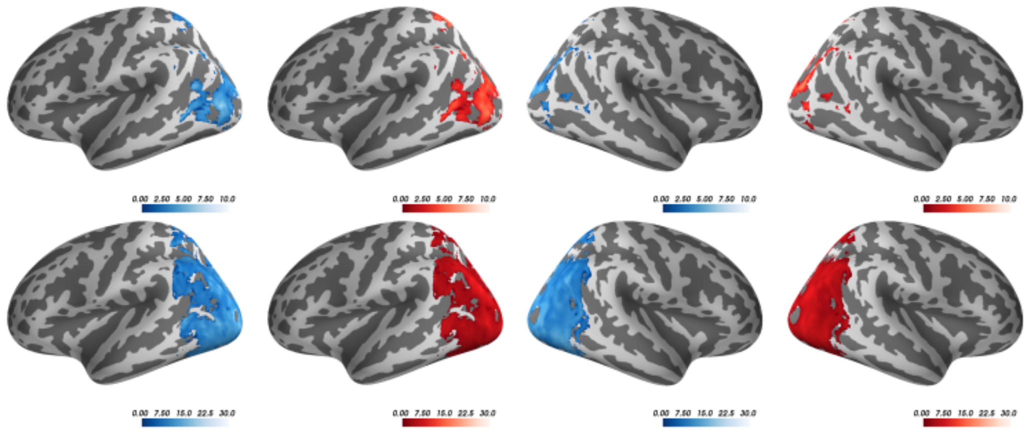

Supplementary Figure 1: Voxels where Euclidean & Mahalanobis(r) overlap (outperforming Pearson). Lateral views of the left and right hemispheres for the GS study (top row) and the NI study (bottom row) displaying t statistics where both the Euclidean measure (blue) and the Mahalanobis(r) measure (red) outperformed the Pearson correlation measure. The t statistics were based on a searchlight analysis of Spearman correlations of each measure with each voxel's SVM confusion matrix (see Materials and Methods). Only displaying t statistics where $p < 0.001$ for paired sample t-tests, TFCE corrected; computed with FSL's randomise function with 5000 permutations, using as a mask the 12 ROIs with best accuracy (see Materials and Methods).

E. Post hoc searchlight analysis

Supplementary Figure 1 presents voxels where both the Euclidean measure and the Mahalanobis(r) measure outperformed Pearson correlation.

References

Bracci, S. and de Beeck, H. O. (2016). Dissociations and associations between shape and category representations in the two visual pathways. *Journal of Neuroscience*, 36(2):432–444.

Jenkinson, M., Beckmann, C. F., Behrens, T. E. J., Woolrich, M. W., and Smith, S. M. (2012). Fsl. *Neuroimage*, 62(2):782–790.

Ledoit, O. and Wolf, M. (2004). A well-conditioned estimator for large-dimensional covariance matrices. *Journal of multivariate analysis*, 88(2):365–411.

Mack, M. L., Preston, A. R., and Love, B. C. (2013). Decoding the brain's algorithm for categorization from its neural implementation. *Current Biology*, 23(20):2023–2027.

Mumford, J. A., Turner, B. O., Ashby, F. G., and Poldrack, R. A. (2012). Deconvolving BOLD activation in event-related designs for multivoxel pattern classification analyses. *Neuroimage*, 59(3):2636–2643.

Pedregosa, F., Varoquaux, G., Gramfort, A., Michel, V., Thirion, B., Grisel, O., Blondel, M., Prettenhofer, P., Weiss, R., and Dubourg, V. (2011). Scikit-learn: Machine learning in Python. *Journal of Machine Learning Research*, 12(Oct):2825–2830.

Pereira, F., Mitchell, T., and Botvinick, M. (2009). Machine learning classi!ers and fMRI: A tutorial overview. *NeuroImage*, 45:S199–S209.

Shepard, R. N. (1987). Toward a universal law of generalization for psychological science. *Science*, 237(4820):1317–1323.
