# Supplementary figures and images for: Measures of Neural Similarity
Source: Comput Brain Behav. 2019 Dec 2;3(4):369–83. doi: 10.1007/s42113-019-00068-5 (PMC7671987; doi:10.1007/s42113-019-00068-5)

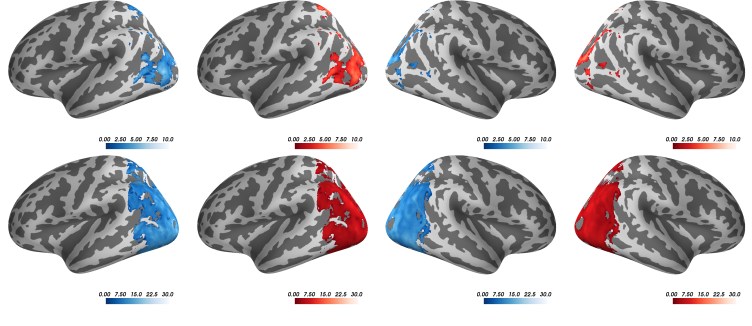

Supplement: Supplementary file 3 — (PNG 120 KB) [file 42113_2019_68_MOESM3_ESM.png]
